# Using the Hepatitis C Virus RNA-Dependent RNA Polymerase as a Model to Understand Viral Polymerase Structure, Function and Dynamics

**DOI:** 10.3390/v7072808

**Published:** 2015-07-17

**Authors:** Ester Sesmero, Ian F. Thorpe

**Affiliations:** Department of Chemistry and Biochemistry, University of Maryland Baltimore County, 1000 Hilltop Circle, Baltimore, MD 21250, USA; E-Mail: sesmero1@umbc.edu

**Keywords:** positive-strand RNA viruses, *Flaviviridae*, conformations

## Abstract

Viral polymerases replicate and transcribe the genomes of several viruses of global health concern such as Hepatitis C virus (HCV), human immunodeficiency virus (HIV) and Ebola virus. For this reason they are key targets for therapies to treat viral infections. Although there is little sequence similarity across the different types of viral polymerases, all of them present a right-hand shape and certain structural motifs that are highly conserved. These features allow their functional properties to be compared, with the goal of broadly applying the knowledge acquired from studying specific viral polymerases to other viral polymerases about which less is known. Here we review the structural and functional properties of the HCV RNA-dependent RNA polymerase (NS5B) in order to understand the fundamental processes underlying the replication of viral genomes. We discuss recent insights into the process by which RNA replication occurs in NS5B as well as the role that conformational changes play in this process.

## 1. Introduction

Polymerases are crucial in the viral life cycle. They have an essential role in replicating and transcribing the viral genome and as a result are key targets for therapies to treat viral infection. A virus may not need to encode its own polymerase depending on where it spends most of its life cycle. Some small DNA viruses that spend all their time in the cell nucleus can make use of the host cell’s polymerases. However, viruses that remain in the cytoplasm do need to encode their own [1].

For viruses that require their own polymerase, most of these enzymes display detectable activity *in vitro* without accessory factors. This is primarily because the sizes of genomes that can be packaged in the viral capsid are limited [1,2]. In addition, some polymerases perform other functions related to viral genome transcription and replication. Examples include the RNA-dependent RNA polymerases from the Flavivirus genus of the Flaviviridae family, retrovirus reverse transcriptases and some viral DNA-dependent polymerases. Flavivirus polymerases have a methyltransferase domain that catalyzes methylations of a 5′-RNA cap [3]. The retrovirus reverse transcriptase has an additional ribonuclease H domain that catalyzes degradation of the RNA strand in the RNA-DNA hybrid during genome replication [4]. Some viral DNA-dependent polymerases have a nuclease domain with proof-reading activity to correct nucleotides incorrectly incorporated during genome synthesis [5].

With regard to copying the viral genome, distinct replication mechanisms are used by different types of viral polymerases. A number of functions must be orchestrated depending on the specific virus in question [1]:

(1) Recognition of the nucleic acid binding site
(2) Coordination of the chemical steps of nucleic acid synthesis
(3) Conformational rearrangement to allow for processive elongation
(3) Termination of replication at the end of the genome

Viral polymerases are often classified into four main categories based on the nature of the genetic material of the virus as follows: RNA-dependent RNA polymerases (RdRps), RNA-dependent DNA polymerases (RdDps), DNA-dependent RNA polymerases (DdRps), and DNA-dependent DNA polymerases (DdDps) [1]. DdDps and DdRps are used for the replication and transcription, respectively, of DNA for both viruses and eukaryotic cells. In contrast, RdDps and RdRps are mainly used by viruses since the host cell does not require reverse transcription or RNA replication. RdDps are employed by retroviruses such as the human immunodeficiency virus (HIV). RdRps are employed by viruses such as Hepatitis C virus (HCV), poliovirus (PV), human rhinovirus (HRV), foot-and-mouth-disease virus (FMDV) and coxsackie viruses (CV) among others. We will primarily focus on RdRps in this review since they are crucial in the replication process of viruses that are important global pathogens.

There are seven classes of viruses according to the Baltimore classification [6] based on the genome type and method of mRNA synthesis. These are associated with the four classes of polymerases specified in the previous paragraph as shown in Table 1.

**Table 1:** Baltimore classification of viruses compared with the classification of viral polymerases based on their targeted genetic material.

Genetic Material	Baltimore Classification	Polymerase Classes	Examples
DNA	ssDNA viruses	DNA dependent DNA polymerases	Human parvovirus B19
	dsDNA viruses	DNA dependent RNA polymerases	Bacteriophage φ29
RNA	(+) ssRNA viruses	RNA dependent RNA polymerases	HCV, PV, West Nile virus
	(−) ssRNA viruses		Influenza
	dsRNA		Bacteriophage φ6
RNA/	ssRNA-rt viruses	RNA dependent DNA polymerases	Retrovirus
DNA	dsDNA-rt viruses		Hepatitis B

## 2. General Structural Features of Viral Polymerases

**Figure 1:**
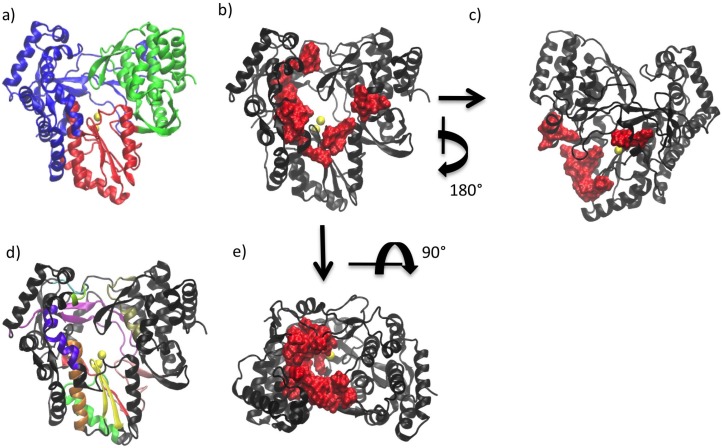
(**a**) Right-hand structure of HCV polymerase (NS5B). Palm, fingers and thumb domains are shown in red, blue and green respectively; (**b**) Duplex channel in NS5B (front of the enzyme); (**c**) NTP channel in NS5B (back of the enzyme); (**d**) Motifs and functional regions of NS5B. Motif A in red, B in orange, C in yellow, D in bright green, E in pink, F in purple and G in cyan. Functional regions: I in light green, II in violet and III in tan; (**e**) Template channel (top view of the enzyme).

The structure of all polymerases resembles a cupped right hand and is divided into three domains referred to as the palm, fingers and thumb (see Figure 1a) [1,7]. This nomenclature is based on an analogy to the structure of the Klenow fragment of DNA polymerase [8]. The palm domain is the most highly conserved domain across different polymerases and is the location of the active site. In contrast, the thumb domain is the most variable. Fingers and thumb domains vary significantly in both size and secondary structure depending on the specific requirements for replication in a given virus (*i.e.*, replicating single- or double-stranded RNA/DNA genomes). The fingers and thumb domains of different polymerases have similar positions with respect to the palm, which contains the active site in which catalytic addition of nucleotides occurs. Changes in the relative positions of the fingers and thumb domains are associated with conformational changes of the polymerase at different stages of replication [7]. Three well-defined channels have been identified on the polymerase, serving as the entry path for template and NTPs (*i.e.*, the template and NTP channels) and exit path for double stranded RNA (dsRNA) product (*i.e.*, the duplex channel) [9,10] (see Figure 1b,c,e).

In the active site, the correct NTP to be added to the daughter strand is selected by Watson-Crick base-pairing with the template base. The selectivity for ribose (rNTP) *vs.* deoxyribose NTPs (dNTP) is regulated by the interaction of the polymerase with the 2′-OH of the NTP. In general, DNA polymerases that incorporate dNTP in the growing daughter strand have a large side chain that prevents binding of an rNTP with a 2′-OH. However, RNA polymerases utilize amino acids with a small side chain and form H-bonds with the 2′-OH of the rNTP. The polymerase active site often binds the correct NTP with 10–1000-fold higher affinity than incorrect NTPs [11].While viral polymerases often have domains in addition to the fingers, palm and thumb that carry out functions related to other aspects of viral genome transcription and replication (see Introduction), this is not the case for the HCV polymerase.

## 3. Conserved Structural Motifs of Viral Polymerases

There are several structural motifs (designated A through G, see Figure 1d) that display varying levels of conservation among the different viral polymerases. Some motifs have been shown to be conserved across all viral polymerases (motifs A to E) while others (motifs F and G) have only been shown to be conserved for the RdRps. High levels of conservation despite the low sequence similarity among polymerases suggests that these motifs have functions that are vital for the action of these enzymes [1,7,9,12,13].

Motifs A and C have been closely studied because they are located in the active site. Motif C includes the GDD amino acid sequence that is the hallmark of RdRps. These conserved residues are bound to the metal ions (Mg^2+^ or Mn^2+^) necessary for catalysis. Motif B contains a consensus sequence of SGxxxT and is located at the junction of the fingers and palm domains [7]. Motif F binds to incoming NTPs and RNA and is situated near the entrance of the RNA template channel. The sequence of this motif is not conserved in *de novo* initiating RdRps such as that present in HCV [14]. These polymerase sequence motifs have also been used to identify new polymerase genes in newly sequenced virus genomes [1]. Further details about the roles of each motif are shown in Table 2. Other regions have also been shown to have fundamental importance in RdRp function and have been named “functional regions”. See Table 3 for a list of the residues included in these regions and the functional roles of each.

**Table 2:** Characteristic sequence motifs in polymerases from prototypical viruses: hepatitis C virus (HCV), poliovirus (PV) and foot-and-mouth-disease virus (FMDV) [7,9,12,15,16].

Conserved Elements		Role	Location	Residues		
				HCV	PV	FMDV
**Motifs**	A	Coordinates Magnesium and selects type of nucleic acid (RNA *vs.* DNA)	Palm	216–227	229–240	236–247
	B	Determines nucleotide choice (rNTP or dNTP)	Palm	287–306	293–312	303–322
	C	Coordinates Magnesium	Palm	312–325	322–335	332–345
	D	Helps accommodate active site NTPs	Palm	332–353	338–362	348–373
	E	Maintains rigidity of secondary structure that is required for relative positioning of thumb and palm domains	Palm	354–372	363–380	374–392
	F	Binds incoming NTPs and RNA	Fingers	132–162	153–178	158–183
	G	Binds primer and template	Fingers	95–99	113–120	114–121
**Functional regions**	I	Binds template	Fingers	91–94	107–112	108–113
	II	Binds template	Fingers	168–183	184–200	189–205
	III	Binds nascent RNA duplex	Thumb	401–414	405–420	416–430

**Table 3:** RdRps virus families and species [1,17].

	Virus family	Representative Species
**(+) ssRNA**	Picornaviradae	Poliovirus (PV)
		Human rhinovirus (HRV)
		Foot-and-mouth-disease virus (FMDV)
		Coxsackie viruses (CV)
		Hepatitis A virus (HAV)
	Caliciviridae	Rabbit hemorrhagic disease virus (RHDV)
		Norwalk virus (NV)
		Sapporo virus
	Togaviridae	Sindbis virus
	Flaviviridae	West Nile virus (WNV)
		Yellow fever virus
		Dengue virus (DENV)
		Japanese encephalitis disease virus (JEV)
		Hepatitis C virus (HCV)
		Bovine viral diarrhea virus (BVDV)
**(−) ssRNA**	Orthomyxoviridae	Influenza virus
	Paramyxoviridae	Measles and mumps viruses
	Bunyaviridae	Hantavirus
	Rhabdoviridae	Rabies virus
	Filoviridae	Ebola and Marburg virus
	Bornaviridae	Borna disease virus
**dsRNA**	Cystoviridae	Bacteriophage ϕ6
	Reoviridae	Reovirus
	Birnaviridae	Fish infectious pancreatic necrosis virus (IPNV)
		Infectious bursal disease virus (IBDV)

## 4. Structural Features of RdRps

RdRps replicate the genomic material in RNA viruses. Many of these viruses are significant public health concerns including HCV, Dengue virus, Japanese encephalitis and yellow fever. For this reason RdRps are key targets for new drugs and it is crucial to understand the mechanisms by which they replicate viral genomes. The fact that there are no mammalian homologs of RdRps [18,19] makes them an optimal drug target because potential therapeutics would tend to selectively affect the viral polymerases without interfering with the function of host polymerases.

Within RdRp encoding viruses there are ssRNA viruses (both + and − sense) and dsRNA viruses (see Table 3). Genome replication in (+) ssRNA viruses takes place in a membrane-bound replication complex [9,20,21]. (+) RNA serves as mRNA and can be translated immediately after entering the cell [1]. Thus, unlike the (−) RNA viruses, (+) RNA viruses do not need to package an RdRp within the virion [22].

The first X-ray structure of an RdRp was generated for Poliovirus (PV) polymerase in 1997 [23]. X-ray structures are currently available from seven families of RdRps. These include (+) RNA viruses: Picornaviridae (PV, HRV, FMDV, CV and HAV), Caliciviridae (RHDV, NV and Sapporo virus) and Flaviviridae (HCV and BVDV) as well as (−) RNA viruses: Orthomyxoviridae (Influenza virus) and dsRNA viruses: Cystoviridae (Bacteriophage ϕ6), Reoviridae (Reovirus and Rotavirus) and Birnaviridae (IBDV). A table listing each NS5B structure currently available in the PDB is included as supporting information. The PDB IDs, a description of each structure and their resolution is provided. Similar information for other viral polymerases is presented in Table 1 and Table 2 of Subissi *et al.* [14].

A characteristic trait of RdRps is the extensive interaction between fingers and palm domains [24]. RdRps have an extension of the fingers domain called the fingertips that connects the fingers and thumb domains to form a fully enclosed active site. The fingertips also contribute to the formation of well-defined template and NTP channels in the front and back of the polymerase, respectively.

RdRps were originally thought to be found uniquely in viruses. However, in 1971 the first eukaryotic RdRp was found in Chinese Cabbage [25]. Later on cellular RdRps were also found in plants, fungi and nematodes [26,27,28]. Cellular RdRps play important roles in both transcriptional and post-transcriptional gene silencing [29]. Although viral and cellular RdRps show little sequence homology, both share the “right hand” shape containing palm, thumb and fingers domains. The palm domain of cellular RdRps is particularly well-conserved and contains four motifs maintained in all polymerases. These facts make it likely that the cellular RdRps share some of the basic mechanistic principles of viral RdRps and that knowledge obtained for viral RdRps may be transferable to cellular RdRps [13].

The Flaviviridae family has been widely studied because many members of this family cause diseases in humans. Within this family there are three genera: Flaviviruses, Hepaciviruses and Pestiviruses (see Table 4). HCV is part of the Hepaciviruses genus and is an important pathogen for which no vaccine is currently available. In explaining recent insights regarding the mechanism by which the HCV RdRp (gene product NS5B) replicates the viral genome, we will make comparisons with other members of the Flaviviridae family. However, we note that some differences may exist, particularly if the other family members are part of a different genus.

**Table 4:** Genera and species of the Flaviviridae family.

Virus Family	Genus	Species
Flaviviridae	Flaviviruses	West Nile virus
		Yellow fever virus
		Dengue virus
		Japanese encephalitis disease virus
	Hepaciviruses	Hepatitis C virus (HCV)
	Pestiviruses	Bovine viral diarrhea virus (BVDV)

## 5. Catalytic Mechanism and Polymerase Reaction Steps

All known polymerases synthesize nucleic acid in the 5′ to 3′ direction [9]. Thus, replication in positive-stranded RNA viruses occurs via a negative-stranded intermediate. The polymerase reaction has three stages: initiation, elongation and termination. For this cycle to take place the polymerase needs to have binding sites for: (a) the template strand; (b) the primer strand or initiating NTP (P-site) and (c) incoming NTP (N-site). The 3′-nucleotide defining the site of initiation is designated “*n*”. Residues at the “*n*” and “*n* + 1” positions of the template define the P-site and N-site.

At the initiation stage, the formation of the first phosphodiester bond is key for polymerization of the nucleotides to begin. To form this phosphodiester bond a hydroxyl group corresponding to a nucleotide 3′-OH is needed. Depending on how this 3′-OH is supplied two mechanisms are differentiated: primer dependent in the case that a primer provides the required hydroxyl group, or primer independent (also called *de novo*) if this hydroxyl group is provided by the first NTP [2]. The variety of mechanisms reflect the adaptation of the viruses to the host cell [1]. The size of the thumb domain seems to define whether a polymerase uses the primer-dependent or *de novo* mechanism. Most viruses in Picornaviridae and Caliciviridae families utilize a primer-dependent mechanism, but exceptions are found, such as noroviruses in the Caliciviridae family, that synthesize the (−) strand *de novo* [30]. In general these enzyme have a small thumb domain that provides a wider template channel to accommodate both template and primer. For this mechanism different primers such as polypeptides, capped mRNAs or oligonucleotides may be used. In contrast, the Flaviviridae family that employs the *de novo* mechanism has a large thumb domain and narrower template channel suited to accommodate only the ssRNA and NTP [1,14]. However, we note that under certain conditions *de novo* polymerases can be induced to become primer dependent [31].

When the *de novo* mechanism is used initiation takes place exactly at the 3′-terminus of the template RNA, so the initiating NTP (the first NTP of the growing strand) is dictated by the template. Both HCV and BVDV from the Flaviviridae family have been observed to require high concentrations of GTP for the initiation of RNA synthesis regardless of the RNA template nucleotide [32,33] which led to the suggestion that GTP may be needed for structural support of the initiating NTP. Harrus *et al.* [34] also suggested that GTP may act as the “initiation platform” and D’Abramo *et al.* [35] pointed out that this GTP may stabilize the interaction between the 3′-end of the template and the priming nucleotide. This stabilizing GTP binds inside the template channel, 6 Å from the catalytic site. It is not incorporated into the nascent RNA strand and is thought to be released from the active site during the elongation stage [9]. We note that another GTP molecule has been reported to bind at the rear of the thumb domain near the fingertips in NS5B. This GTP has been suggested to play a role in activating *de nov*o initiation or in allosterically regulating the conformational changes needed for replication [36].

**Figure 2:**
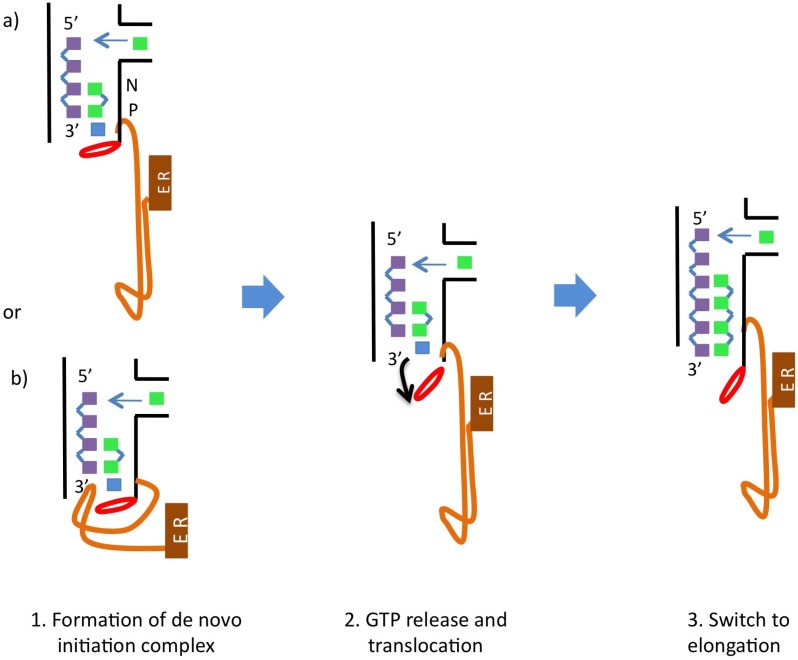
Schematic describing de novo initiation in Hepaciviruses and Pestiviruses. Note that Flaviruses do not anchor their C-terminus in the Endoplasmatic Reticulum (ER). This figure was generated by incorporating the descriptions provided by both Appleby *et al.* [35] and Choi [1]. The linker and C-terminal anchor are shown in orange as one contiguous element. The β-flap is colored red (as in Figure 3), the template strand in purple, the growing strand in green, the stabilizing GTP in blue and the Endoplasmic Reticulum (ER) in brown. The “N” and “P” indicate where the N-site (nucleotide-site) and P-site (priming-site) are. These correspond to the positions of the growing strand that bind to residues “*n*” and “*n* + 1” of the template strand respectively.

Because base-pairing alone is insufficient to stabilize the dinucleotide product in the “P-site”, specialized structural elements are employed [13]. Besides the stabilizing GTP there is also a polymerase structural initiation platform, the so-called β-flap (residues 443–454). This β-flap likely supports the stabilizing GTP but would need to move out of the way in the elongation phase to allow the dsRNA product to exit [1,9,34]. Other researchers have suggested the C-terminal linker (residues 531 to 570) also plays a regulatory role in the initiation stage of replication by acting as a buttress during the initiation stage and moving out of the template channel in a similar way as the β-flap in order to allow egress of the double stranded RNA [14,37] (see Figure 2).

**Figure 3:**
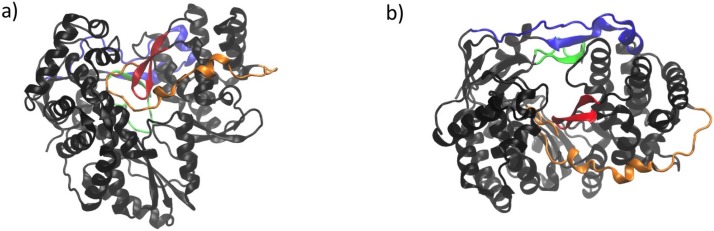
NS5B structure with characteristic elements highlighted. (**a**) Front view and (**b**) top view. The linker is shown in orange, the β-flap in red. The fingertips are shown in blue (the delta 1 loop) and green (the delta 2 loop).

An advantage of the primer-dependent mechanism is that a stable elongation complex is formed more easily. There is limited abortive cycling, if any, and no requirement for large conformational rearrangements [13]. In contrast, for the *de novo* mechanism the first dinucleotide is not sufficiently stable and an initiation platform is needed to provide additional stabilization. This reduced stability sometimes results in abortive cycling for the *de novo* mechanism. However, an advantage of the *de novo* mechanism is that no additional enzymes are needed to generate the primer [38].

After the template and primer or initiating NTP are bound to the enzyme, the steps required for single-nucleotide addition are [1]:

(1) incorporation of the incoming NTP into the growing daughter strand by formation of the phosphodiester bond
(2) release of pyrophosphate
(3) translocation along the template.

These three steps are repeated cyclically during elongation until the full RNA strand is replicated.

**Figure 4:**
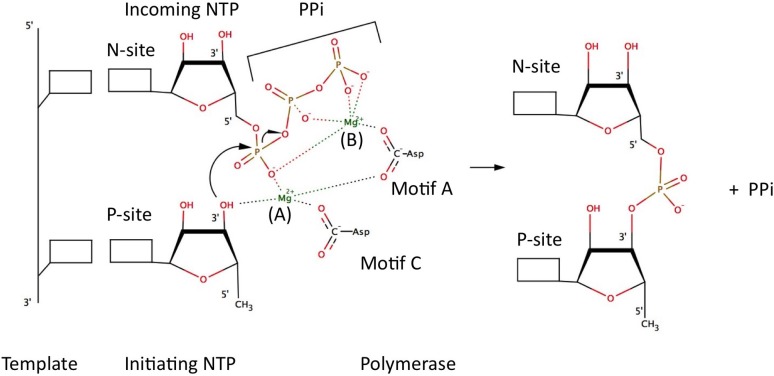
Two metals ions mechanism in RdRps. The squares represent the bases that are part of the nucleotides. This figure is inspired by a similar figure from Choi *et al.* [1].

In order to facilitate nucleotide addition, all polymerases have two metal ions (Mg^2+^ or Mn^2+^) in the active site bound to two conserved aspartic acid residues. These metal ions have been shown to be essential for catalysis via the so-called “two metal ions” mechanism. This mechanism was proposed by Steiz in 1998 [39] and is as follows: the incoming NTP binds to metal ion B that orients the NTP in the active site and that may contribute to charge neutralization during catalysis. Metal ion B coordinates to the β- and γ-phosphate groups of the incoming NTP as well as the aspartic acid residue in motif A. Once the nucleotide is in place, the second divalent cation (Metal ion A) coordinates to the initiating NTP, lowering the pKa of the 3′-OH and facilitating nucleophilic attack on the α-phosphate. This then leads to formation of the phosphodiester bond and the release of pyrophosphate (PPi). Metal ion A coordinates to the α-phosphate group of the incoming NTP, the 3′-OH of the priming NTP and the aspartic acid residue in motif C (see Figure 4). Both metal ions stabilize the charge and geometry of the phosphorane pentavalent transition state during the nucleotidyl transfer reaction [1,13,38].

The switch to elongation requires a major conformational change in the polymerase structure. Both the β-flap and the linker need to be displaced and an opening of the enzymatic core occurs. This open conformation may be one of the factors that enables a higher processivity in the elongation stage compared to the initiation stage (for more detailed information about this change in conformation see Section 5 below).

Little is known about the termination of RNA synthesis. It has been suggested that the polymerase may simply fall off the end of the template once the complementary strand has been synthesized [14,40]. It is important to note that RNA synthesis by NS5B is error-prone due to the lack of proofreading activity of the RdRp enzymes. The mutation rates are estimated to be on the order of one mutation per 10^3^–10^7^ nucleotides resulting in approximately one error per replicated genome [1,41]. In contrast the mutation rate in *E. coli*, where cellular polymerases benefit from error-correcting mechanisms, is on the order of one mutation per 10^9^–10^10^ nucleotides [1]. The large error rate results in the high genetic variability of the HCV viruses and provides a molecular basis for the rapid development of resistance to therapies.

## 6. NS5B Conformational Changes during the Replication Cycle

One characteristic unique to viral RdRps is their “closed-hand” shape. This terminology started to be used because their X-ray structures appear to be more closed than the previously characterized DdDps, DdRps and RTs (called “open-hand”) [7,14,38,42]. This “closed-hand” shape is characterized by the fingertips region, a hallmark of RdRps, that connects the fingers and palm domains on the back of the enzyme as well as by the so-called β-flap on the front of the enzyme (see Figure 3). The latter is specific to the Flaviviridae RdRps while the linker, or a variation of it, is common to most of the *de novo* initiating RdRps [40] (note, however, that it is not found in the Flavivirus RdRps). The linker (residues 531 to 570) connects the NS5B catalytic core (residues 1 to 530) with the C-terminus transmembrane anchor (residues 571 to 591). These last twenty-one C-terminal residues seem not to influence RNA synthesis *in vitro* [40]. Given that these residues are very hydrophobic, their removal facilitates expression and purification of the enzyme. Thus, most biochemical and all structural studies have been carried out with the so-called NS5B Δ21 enzyme variant in which these residues have been removed.

Most of the NS5B structures that have been reported are thought to be in the closed conformation. However, it has been observed that *de novo* initiation by NS5B *in vitro* does not only occur at the 3′ end of the template but also can take place at internal template sites [43,44] and on circular templates [31]. These facts suggest that in solution there is an equilibrium between the closed and open conformations. The existence of the open conformation is supported by the structure of NS5B from genotype 2a NS5B, [45] as well as the structure recently published by Mosley *et al.* [46]. The latter contains a variant of NS5B that lacks the β-flap in complex with primer-template RNA. Molecular dynamics simulations of Davis *et al.* [47] also indicate the occurrence of open NS5B conformations.

The closed conformation is thought to represent the initiation state of the polymerase. In this conformation the catalytic core only provides sufficient space for a single-stranded RNA template and the nucleotides required for *de novo* initiation of RNA synthesis, but is not wide enough to accommodate double-stranded RNA [40]. To transition to elongation a major conformational change is needed so the nascent RNA can egress. Primer-dependent RdRps undergo less dramatic conformational changes than *de novo*-initiating RdRps [14] because the thumb domain of primer-dependent RdRps is smaller, leaving enough room for the dsRNA product to exit. Transitioning to elongation in *de novo*-initiating RdRps thus requires the adoption of an open conformation [34,40,46,48]. To arrive at the open conformation the β-flap would need to be moved out of the way, the stabilizing GTP should unbind and also a rotation of the thumb domain should take place. This would position it further from the center of the enzyme, increasing the size of the template and duplex channels so the dsRNA can exit the enzyme [34,48]. If the C-terminal linker does act as an initiation platform together with the β-flap, this element would also need to move away from the template channel in the transition to elongation as described by Appleby *et al.* [37] (see Figure 2). It is worth noting that conformational changes have been reported in several RdRp structures [45,46,49]. These findings suggest that these enzymes exhibit considerable conformational variability, which is similar to observations made for other polymerases [50,51].

## 7. NS5B Inhibitors and Mechanisms of Action

**Figure 5:**
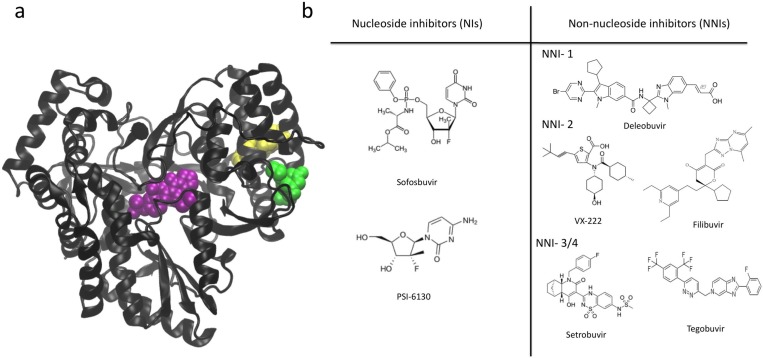
NS5B inhibitors. (**a**) The three allosteric sites of NS5B are highlighted with space filling representations of inhibitors that bind in these locations. Thumb site 1 (NNI-1) in yellow, thumb site 2 (NNI-2) in green, palm sites (NNI-3/4) in purple; (**b**) chemical structures of NIs and NNIs that are in clinical trials or have already been approved [52,53,54,55,56,57,58,59].

There are two main classes of NS5B inhibitors: nucleoside inhibitors (NIs) and non-nucleoside inhibitors (NNIs) (see Figure 5). NIs bind in the active site and generally act as non-obligate terminators of RNA synthesis after being incorporated into the newly produced RNA strand. The advantages of NIs are that they have shown stronger antiviral activity, are able to inhibit multiple HCV genotypes and have a higher barrier to the emergence of drug resistance [48]. However, they have the potential to also affect host polymerases since they interact with an active site that has similar features among diverse types of polymerases. Sofosbuvir, the drug most recently approved for HCV treatment is in this group. NNIs are allosteric inhibitors that bind to sites other than the active site. NNIs are also promising, though they have not yet been used in a clinical setting. NNIs are attractive for use in future anti-HCV therapies due to the decreased likelihood that they will exhibit nonspecific side effects compared to NIs. However, HCV is more likely to become resistant to these inhibitors because there is typically not strong evolutionary pressure to maintain the amino acid sequence of NNI binding sites. We focus on NNIs in this review because the role of NIs as terminators of RNA synthesis is well understood. In contrast, although many structures with NNIs bound have been solved, their mechanism of action still remains to be elucidated.

**Figure 6:**
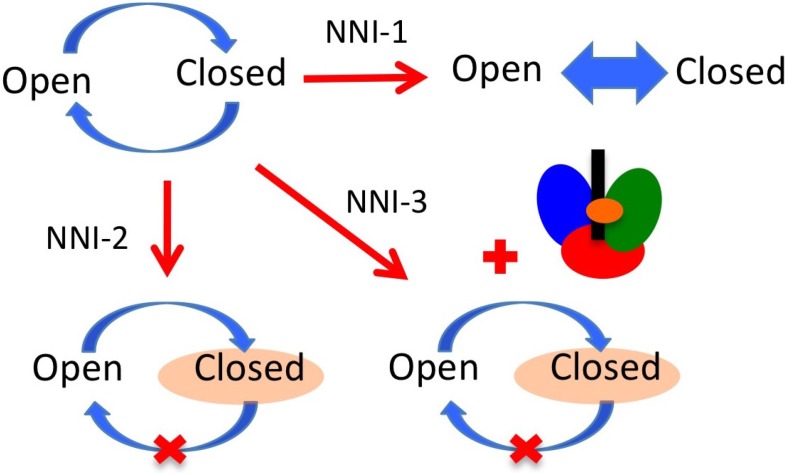
Mechanisms of inhibition for NNIs. NS5B must transition between open and closed states to perform replication (upper left). NNI-1 inhibitors have been observed to reduce enzyme stability. NNI-2 inhibitors have been shown to reduce conformational sampling, confining the enzyme in closed conformations. NNI-3 inhibitors mainly block access of the RNA template but also induce some restriction of conformational sampling. The RNA template is represented as a black rectangle and the inhibitor as an orange ellipse.

Four NNI sites have been identified: two in the thumb (NNI-1 and NNI-2) and two in the palm (NNI-3 and NNI-4) (see Figure 5). Brown and Thorpe [60] provide evidence that NNI-3 and NNI-4 are likely to be distinct regions within a single large pocket rather than two individual pockets. For this reason we use the nomenclature NNI-3/4 to denote both of these partially overlapping sites. Due to the fact that there are multiple distinct allosteric sites it may be possible to use multiple NNIs in combination with each other or with NIs in the effort to overcome resistance. NNIs are thought to inhibit NS5B by affecting the equilibrium distribution of conformational states required for normal catalytic activity of the enzyme [13,47]. Most of the NNIs that bind to the palm domain have been found to stabilize the β-flap via critical interactions with Tyr448 [61], fixing it in the closed, initiation-appropriate conformation and preventing these residues from moving out to allow the RNA double helix to egress [46]. NNI-2 ligands have also been suggested to prevent the occurrence of important conformational changes in NS5B [16,45,62]. Some studies have suggested that palm NNIs inhibit initiation while thumb NNIs inhibit an early phase of replication that occurs after initiation but before elongation starts [63,64,65,66]. Thus, the different allosteric sites may display distinct modes of action. Davis *et al.* [46] studied the mechanism of inhibition of allosteric inhibitors in the different allosteric sites. They found that inhibitors in the NNI-1 pocket seem to prevent enzyme function by reducing its overall stability and preventing it from stably adopting functional conformations. In contrast, NNI-2 inhibitors seem to reduce conformational sampling, preventing the transitions between conformational states that are required for NS5B to function. NNI-3 inhibitors were also observed to restrict conformational sampling, though the dominant mode of action of these molecules was predicted to result from blocking access of the RNA template (see Figure 6).

This may facilitate their use in combination therapies by degrading complementary functionalities in the enzyme. Understanding the molecular mechanisms by which small molecules in general and NNIs in particular inhibit the function of NS5B is essential for rationally design NS5B inhibitors. Such molecules may ultimately serve as a basis for more efficacious or cost-effective HCV therapies, either individually or in combination.

One informative example that illustrates the useful interplay between determining the roles of structural and functional elements of NS5B and understanding the efficacy of NNIs is provided by recent studies of Gilead pharmaceuticals. Boyce *et al.* [67] assessed the activities and biophysical properties of a number of NS5B variants using mutations and deletions in the enzyme C-terminus and β-flap, in concert with challenging the enzyme using diverse NNIs. Their observations suggest that ligands which bind to NNI-2 exhibit a unique inhibitory mechanism relative to other NNIs. Boyce *et al.* [67] discovered that NNI-2 ligands are most effective when both the C-terminus and β-flap of the enzyme are present. These inhibitors were found to stabilize NS5B in a closed conformation, consistent with simulation studies by Davis *et al.* [16,47]. Boyce *et al.* [67] found that interactions between the C-terminus and the β-flap were required for inhibition, but not for ligand binding. These authors determined that NNI-2 inhibitors exhibited decreased efficacy for truncated NS5B variants and suggested that while the C-terminus and β-flap do not alter the intrinsic interactions of NNI-2 ligands with the enzyme, they do play an important role in propagating the allosteric effects that result from inhibitors binding to distant enzyme locations. This finding is consistent with mutational data for NNI-2 inhibitors, which map viral resistance mutations to areas around the β-flap [68].

Simulation studies by Davis and Thorpe suggest that the enzyme C-terminus reduces conformational sampling in NS5B, likely eliminating transitions between the closed and open conformations necessary for the initiation and elongation phases of replication respectively [69]. These observations predict that enzymes without C-terminal residues should display increased activity, consistent with the findings of Boyce *et al.* [67]. Other studies from Davis *et al.* [16,47] indicate that an NNI-2 ligand can restrict conformational sampling even if the C-terminus is absent, stabilizing the enzyme in a very closed state. One might expect that this property could account for the inhibitory action of NNI-2 ligands without needing to invoke a role for the C-terminus as suggested by Boyce *et al.* [67]. However, there are several important considerations to be noted. First, the simulation studies examine the impact of binding a ligand to the enzyme and do not directly probe inhibition. The studies of Boyce *et al.* [67] indicate that binding affinities of NNI-2 ligands are not a good proxy for inhibition efficacy. Thus, observations in the simulation studies may not be explicitly linked to allosteric inhibition. Another consideration is that the simulation studies were not carried out with both the inhibitor and the C-terminus present. It is possible that conformational restriction of the enzyme in the presence of both entities would be even more dramatic, consistent with the enhanced inhibition in Boyce *et al.* [67] measured in the presence of the C-terminus. Finally, different ligands were employed in each study and it could be that distinct inhibitors elicit different effects even though they bind to the same location. There is evidence that different NNI-2 ligands are able to alter the conformational distribution of NS5B to different extents [47]. Thus, it is possible that the enzyme C-terminus is only required for observing the inhibitory effects of certain NNI-2 ligands.

In contrast to NNI-2, Boyce *et al.* [67] observed that the potency of NNI-1 ligands was not affected by the presence of the C-terminus or β-flap. This observation suggests that these ligands possess a completely different mechanism of action compared to NNI-2 inhibitors. These authors noted that the presence of NNI-1 ligands lowered the melting temperature of NS5B, consistent with decreased stability of the enzyme. The decrease of NS5B stability in the presence of NNI-1 ligands was noted as well in other studies [70,71]. This finding is also consistent with results from simulations of Davis *et al.* [47] that suggest NNI-1 and NNI-2 ligands have distinct modes of action. In contrast to the stabilizing effect of NNI-2 ligands, it was observed that an NNI-1 ligand destabilized conformational sampling in NS5B, preventing the enzyme from stably occupying functional conformational states.

With regard to palm inhibitors, Boyce *et al.* [67] observed that such ligands display larger dissociation constants in NS5B constructs for which C-terminal residues were deleted, suggesting that the C-terminus facilitates binding to palm sites. Palm site inhibitors also demonstrate decreased potency in these deletion constructs, indicating that the C-terminus is needed for both binding and inhibition. In simulation studies Davis *et al.* [47] observed that NNI-3 ligands were able to bind to the enzyme without the C-terminus present and also restricted conformational sampling of NS5B in a similar manner to NNI-2 ligands. However, the conformations sampled when ligands were bound to NNI-3 tended to be more open in general than those induced by an NNI-2 ligand. These conformations may perturb the replication cycle to a reduced extent compared to NNI-2 or NNI-1 ligands. It is possible that in the presence of the C-terminus NNI-3 ligands elicit more dramatic changes in conformational sampling. Nonetheless, the authors concluded that the dominant inhibitory effect of palm ligands is likely due to direct obstruction of the RNA template channel (thus preventing the template from accessing the active site) rather than conformational restriction. This observation is consistent with previous predictions [72].

The findings of Boyce *et al.* [67] are important because they indicate the enzyme C-terminus plays a crucial role in modulating the efficacy of NNIs. The likely molecular basis of this observation can be readily understood by considering the schematic shown in Figure 2. In this figure it is apparent that the C-terminus acts as a “stopper” in the template channel, preventing elongation of the nascent RNA strand. Thus, both the C-terminus and β-flap need to be removed from the template channel before elongation can proceed. If the C-terminus is not present, the template channel cannot be effectively blocked and replication is less likely to be affected by presence of the inhibitor. This is a quite interesting result, as it points to the limitations of some inhibitor studies that may have been carried out *in vitro* using enzyme variants without the C-terminus. It is likely that any ligands employing the inhibitory mechanisms described by Boyce *et al.* [67] would not be identified in such studies. Thus, the role of NS5B regulatory elements in strongly modulating the efficacy of inhibitors must be taken into account when assessing ligand potency.

Studies such as those of Boyce *et al.* [67] or Davis and colleagues [16,47,69] may be useful to understand the differing susceptibility of different NS5B variants (and thus different HCV strains or genotypes) to the presence of diverse inhibitors. For example, in some viral genotypes the C-terminus might interact more strongly with the template channel than in others. One would anticipate that NNI-2 ligands would be more effective in inhibiting such enzyme variants. The studies reviewed in this article indicate that understanding the structure and function of NS5B provides powerful insight into the molecular mechanisms governing inhibition of this enzyme and the functional properties of other RdRps. For example, recent structural studies of the Influenza virus polymerase reveal a β-flap element similar to that which modulates the activity of NS5B and which may adopt a similarly important role in these enzymes [73,74]. We note that simulation studies are particularly helpful in this regard by allowing molecular mechanisms underlying the observed structure-function relationships to be elucidated [16,47,69].

Understanding the molecular mechanisms involved in inhibition by NNIs could facilitate the design and deployment of these molecules. The insights acquired may also be transferable to other polymerases to better understand the relationship between structure, function and dynamics in these enzymes. Due to the fact that individual NNIs can have distinct sites of binding, it should be possible to combine multiple NNIs such that their total inhibitory effect is enhanced relative to applying any given inhibitor on its own [60]. It may be beneficial to target complementary activities or distinct conformational states of the enzyme with an array of small molecules to degrade a wide spectrum of NS5B functionality in a therapeutic context. For example, it is possible that a large fraction of NS5B exists within the host cell in an auto-inhibited state with the C-terminus occupying the template channel. In this way, the virus can avoid negatively perturbing the host cell and facilitate evasion of the host immune response. One could envision targeting both actively replicating and auto-inhibited NS5B molecules with different inhibitors in order to more effectively degrade intracellular enzyme activity.

## 8. Summary

*Flaviviridae* viruses are (+) RNA viruses with RdRp polymerases that utilize the *de novo* mechanism for initiation. While *Flaviviridae* polymerases possess elements common to other RdRps such as the fingertips region, they are also unique in possessing the β-flap that may be used as an initiation platform during genome replication.

The important pathogen HCV is a member of the *Flaviviridae* family within the *Hepacivirus* genus and employs NS5B as the RdRp that replicates its genome. There are two key steps involved in the replication process: (1) the formation of the initial dinucleotide and (2) the transition from initiation to processive elongation. Structural elements of NS5B that likely have a crucial role in these steps are the C-terminal linker and the β-flap (see Figure 3). Initiation is also facilitated by the so-called “stabilizing GTP” in the active site (see Figure 2). Finally, a conformational change involving movement of the thumb and fingers domains to position them further apart has been observed to accompany the transition from initiation to elongation, resulting in an open-hand conformation. The linker and the β-flap may have dual roles: (1) acting as initiation platforms to stabilize formation of the first dinucleotide and (2) regulating the transition to elongation. These structural elements can prevent the enzyme from moving to the elongation stage and must be displaced to allow for processive elongation to take place.

Thus, the available evidence suggests that NS5B possesses an intrinsic capacity to be regulated via allosteric effectors including NNIs, the β-flap and the C-terminal linker. In addition, the role of these effectors seems to be strongly modulated by the specific context of the interaction. Understanding how these structural elements govern enzyme activity and how they interface with inhibitors is important for understanding the molecular mechanisms of allosteric inhibition in NS5B. Such knowledge paves the way for rational design of inhibitors and combination therapies both for NS5B and for the polymerases to which these insights can be generalized. This information may also be useful in designing enzymes with attenuated activity, as would be required if one sought to develop a strain of HCV that could serve as the basis for a vaccine. Attenuating HCV by degrading the activity of NS5B is one strategy that could prove useful in this regard. One potential drawback to such efforts is the high mutation rate of HCV that results from the error-prone nature of NS5B. However, it is possible that one could circumvent this issue by generating a polymerase that not only possesses reduced efficacy, but also displays increased fidelity and thus faithfully replicates the viral genome.

Viral polymerases and, specifically, RdRps share many common structural, functional and dynamic features. Thus, the knowledge obtained in understanding how NS5B functions may be transferable to polymerases from closely related viruses such as Dengue or West Nile virus, or even to other more distantly related polymerases such as reverse transcriptase from HIV and 3D-pol from poliovirus.

## References

[1] Choi K.H. (2012). Viral polymerases. Viral Molecular Machines.

[2] Ortin J., Parra F. (2006). Structure and function of RNA replication. Annu. Rev. Microbiol..

[3] Zhou Y., Ray D., Zhao Y., Dong H., Ren S., Li Z., Guo Y., Bernard K.A., Shi P.-Y., Li H. (2007). Structure and function of flavivirus ns5 methyltransferase. J. Virol..

[4] Zhou D., Chung S., Miller M., Grice S.F.J.L., Wlodawer A. (2012). Crystal structures of the reverse transcriptase-associated ribonuclease h domain of xenotropic murine leukemia-virus related virus. J. Struct. Biol..

[5] Knopf C. (1998). Evolution of viral DNA-dependent DNA polymerases. Virus Genes.

[6] Baltimore D. (1971). Expression of animal virus genomes. Bacteriol. Rev..

[7] Shatskaya G.S. (2013). Structural organization of viral RNA-dependent RNA polymerases. Biochemistry.

[8] Ollis D.L., Brick P., Hamlin R., Xuong N.G., Steitz T.A. (1985). Structure of large fragment of *Escherichia coli* DNA polymerase i complexed with dtmp. Nature.

[9] McDonald S.M. (2013). RNA synthetic mechanisms employed by diverse families of RNA viruses. WIREs RNA.

[10] Ferrer-Orta C., Verdaguer N., Cameron C., Gotte M., Raney K.D. (2009). RNA virus polymerases. Viral Genome Replication.

[11] Gao G., Orlova M., Georgiadis M.M., Hendrickson W.A., Goff S.P. (1997). Conferring RNA polymerase activity to a DNA polymerase: A single residue in reverse transcriptase controls substrate selection. Proc. Natl. Acad. Sci. USA.

[12] Cameron C.E., Moustafa I.M., Arnold J.J. (2009). Dynamics: The missing link between structure and function of the viral RNA-dependent RNA polymerase?. Curr. Opin. Struct. Biol..

[13] Ng K.K.-S., Arnold J.J., Cameron C.E. (2008). Structure and Function Relationships Ammong RNA-Dependent RNA Polymerases.

[14] Subissi L., Decroly E., Selisko B., Canard B., Imbert1 I. (2014). A closed-handed affair: Positive-strand RNA virus polymerases. Future Virol..

[15] Moustafa I.M., Shen H., Morton B., Colina C.M., Cameron C.E. (2011). Molecular dynamics simulations of viral RNA polymerases link conserved and correlated motions of functional elements to fidelity. J. Mol. Biol..

[16] Davis B., Thorpe I.F. (2013). Thumb inhibitor binding eliminates functionally important dynamics in the hepatitis c virus RNA polymerase. Proteins Struct. Funct. Bioinform..

[17] International Committee on Taxonomy of Viruses. http://www.Ictvonline.Org.

[18] Gong J., Fang H., Li M., Liu Y., Yang K., Xu W. (2009). Potential targets and their relevant inhibitors in anti-influenza fields. Curr. Med. Chem..

[19] Malet H., Masse N., Selisko B., Romette J.L., Alvarez K., Guillemot J.C., Tolou H., Yap T.L., Vasudevan S., Lescar J. (2008). The flavivirus polymerase as a target for drug discovery. Antivir. Res..

[20] Welsch S., Miller S., Romero-Brey I. (2009). Composition and three-dimensional architecture of the dengue virus replication and assembly sites. Cell Host Microbe.

[21] Hsu N.Y., Ilnytska O., Belov G. (2010). Viral reorganization of the secretory pathway generates distinct organelles for RNA replication. Cell.

[22] Zuckerman A.J., Baron S. (1996). Hepatitis viruses. Medical Microbiology.

[23] Hansen J.L., Long A.M., Schultz S.C. (1997). Structure of the RNA-dependent RNA polymerase of poliovirus. Structure.

[24] Lindenbach B.D., Tellinghuisen T.L., Cameron C., Gotte M., Raney K.D. (2009). Hepatitis C virus genome replication. Viral Genome Replication.

[25] Astier-Manifacier S., Cornuet P. (1971). RNA-dependent RNA polymerase in chinese cabbage. Biochim. Biophys. Acta.

[26] Boege F., Heinz L.S. (1980). RNA-dependent RNA polymerase from healthy tomato leaf tissue. FEBS Lett..

[27] Cogoni. C., Macino G. (1999). Gene silencing in neurospora crassa requires a protein homologous to RNA-dependent RNA polymerase. Nature.

[28] Smardon A., Spoerke J.M., Stacey S.C., Klein M.E., Mackin N., Maine E.M. (2000). Ego-1 is related to RNA-directed RNA polymerase and functions in germ-line development and RNA interference in c. Elegans. Curr. Biol..

[29] Maida Y., Masutomi K. (2011). RNA-dependent RNA polymerases in RNA silencing. Biol. Chem..

[30] Rohayem J., Robel I., Jager K., Scheffler U., Rudolph W. (2006). Protein-primed and *de novo* initiation of RNA synthesis by norovirus 3dpol. J. Virol..

[31] Ranjith-Kumar C.T., Kao C.C. (2006). Recombinant viral rdrps can initiate RNA synthesis from circular templates. RNA.

[32] Luo G., Hamatake R.K., Mathis D.M., Racela J., Rigat K.L., Lemm J., Colonno R.J. (2000). *De novo* initiation of RNA synthesis by the RNA-dependent RNA polymerase (ns5b) of hepatitis C virus. J. Virol..

[33] Kao C.C., Vecchio A.M.D., Zhong W. (1999). *De novo* initiation of RNA synthesis by a recombinant flaviviridae RNA-dependent RNA polymerase. Virology.

[34] Harrus D. (2010). Further insights into the roles of GTP and the C terminus of the hepatitis C virus polymerase in the initiation of RNA synthesis. J. Biol. Chem..

[35] D’Abramo C.M., Deval J., Cameron C.E., Cellai L., Gotte M. (2006). Control of template positioning during de novo initiation of RNA synthesis by the bovine viral diarrhea virus NS5B polymerase. J. Biol. Chem..

[36] Bressanelli S. (2002). Structural analysis of the hepatitis C virus RNA polymerase in complex with ribonucleotides. J. Virol..

[37] Appleby T.C., Perry J.K., Murakami E., Barauskas O., Feng J., Cho A., Fox D., Wetmore D.R., McGrath M.E., Ray A.S. (2015). Structural basis for RNA replication by the hepatitis C virus polymerase. Science.

[38] Van Dijk A.A., Makeyev E.V., Bamford D.H. (2004). Initation of viral RNA-dependent RNA polymerization. J. Gen. Virol..

[39] Steitz T. (1998). A mechanism for all polymerases. Nature.

[40] Lohmann V. (2013). Hepatitis C Virus: From Molecular Virology to Antiviral Therapy.

[41] Drake J.W. (1991). A constant rate of spontaneous mutation in DNA-based microbes. Proc. Natl. Acad. Sci. USA.

[42] Ferrer-Orta C., Arias A., Escarmi C., Verdaguer N. (2006). A comparison of viral RNA-dependent RNA polymerases. Curr. Opin. Struct. Biol..

[43] Binder M., Quinckert D., Bochkarova O., Klein R., Kezmic N., Bartenschalager R., Lohmann V. (2007). Identification of determinants involved in initatiation of hepatitis c virus RNA synthesis by using intergenotipic chimeras. J. Virol..

[44] Shim J.H., Larson G., Hong J.Z. (2002). Selection of 3′ template bases and initatiting nucleotides by hepatitis c virus RNA by and ago2-miR-122 complex. Proc. Natl. Acad. Sci. USA.

[45] Biswal B.K., Cherney M.M., Wang M., Chan L., Yannopoulos C.G., Bilimoria D., Nicolas O., Bedard J., James M.N. (2005). Crystal structures of the RNA-dependent RNA polymerase genotype 2A of hepatitis C virus reveal two conformations and suggest mechanisms of inhibition by non-nucleoside inhibitors. J. Biol. Chem..

[46] Mosley R.T. (2012). Structure of hepatitis C virus polymerase in complex with primer- template RNA. J. Virol..

[47] Davis B.C., Brown J.A., Thorpe I.F. (2015). Allosteric inhibitors have distinct effects, but also common modes of action, in the hcv polymerase. Biophys. J..

[48] Caillet-Saguy C., Lim S.P., Shi P.-Y., Lescar J., Bressanelli S. (2014). Polymerases of hepatitis C viruses and flaviviruses: Structural and mechanistic insights and drug development. Antivir. Res..

[49] Choi K.H., Groarke J.M., Young D.C., Kuhn R.J., Smith J.L., Pevear D.C., Rossmann M.G. (2004). The structure of the RNA-dependent RNA polymerase from bovine viral diarrhea virus establishes the role of GTP in de novo initiation. Proc. Natl. Acad. Sci. USA.

[50] Rothwell P.J., Waksman G. (2005). Structure and mechanism of DNA polymerases. Adv. Protein Chem..

[51] Doublie S., Sawaya M.R., Ellenberger T. (1999). An open and closed case for all polymerases. Structure.

[52] Wendt A., Adhoute X., Castellani P., Oules V., Ansaldi C., Benali S., Bourliere M. (2014). Chronic hepatitis c: Future treatment. Clin. Pharmacol..

[53] Larrey D., Lohse A.W., de Ledinghen V., Trepo C., Gerlach T., Zarski J.P., Tran A., Mathurin P., Thimme R., Arasteh K. (2012). Rapid and strong antiviral activity of the non-nucleosidic NS5B polymerase inhibitor BI 207127 in combination with peginterferon α 2a and ribavirin. J. Hepatol..

[54] Jacobson I., Pockros P.J., Lalezari J., Lawitz E., Rodriguez-Torres M., DeJesus E., Haas F., Martorell C., Pruitt R., Purohit V. (2010). Virologic response rates following 4 weeks of filibuvir in combination with pegylated interferon α-2a and ribavirin in chronically-infected HCV genotype-1 patients. J. Hepatol..

[55] Rodriguez-Torres M., Lawitz E., Conway B., Kaita K., Sheikh A.M., Ghalib R., Adrover R., Cooper C., Silva M., Rosario M. (2010). Safety antiviral activity of the HCV non-nucleoside polymerase inhibitor VX-222 in treatment-naive genotype 1 HCV-infected patients. J. Hepatol..

[56] Lawitz E., Rodriguez-Torres M., Rustgi V.K. (2010). Safety and antiviral activity of ana 598 in combination with pegylated interferon α-2a plus ribavirin in treatment-naive genotype 1 chronic HCV patients. J. Hepatol..

[57] Lawitz E., Jacobson I., Godofsky E., Foster G.R., Flisiak R., Bennett M., Ryan M., Hinkle J., Simpson J., McHutchison J. (2011). A phase 2b trial comparing 24 to 48 weeks treatment with tegobuvir (GS-9190)/PEG/RBV to 48 weeks treatment with PEG/RBV for chronic genotype 1 HCV infection. J. Hepatol..

[58] Gane E.J., Stedman C.A., Hyland R.H. (2013). Nucleotide polymerase inhibi- tor sofosbuvir plus ribavirin for hepatitis C. N. Engl. J. Med..

[59] Wedemeyer H., Jensen D., Herring R. (2012). Efficacy and safety of mericitabine in combination with PEG-IFN α-2a/RBV in G1/4 treatment naive HCV patients: Final analysis from the propel study. J. Hepatol..

[60] Brown J.A., Thorpe I.F. (2015). Dual allosteric inhibitors jointly modulate protein structure and dynamics in the hepatitis c virus polymerase. Biochemistry.

[61] Pfefferkorn J.A. (2005). Inhibitors of hcv ns5b polymerase. Part 1: Evaluation of the southern region of (2*Z*)-2-(benzoylamino)-3-(5-phenyl-2-furyl)acrylic acid. Bioorg. Med. Chem. Lett..

[62] Wang M. (2003). Non-nucleoside analogue inhibitors bind to an allosteric site on hcv ns5b polymerase. Crystal structures and mechanism of inhibition. J. Biol. Chem..

[63] Ontoria J.M., Rydberg E.H., Carfi A. (2009). Identification and biological evaluation of a series of 1*H*-benzo[*de*]isoquinoline-1,3(2*H*)-diones as hepatitis C virus NS5B polymerase inhibitors. J. Med. Chem..

[64] Nyanguile O., Pauwels F., van den Broeck W., Boutton C.W., Quirynen L., Ivens T., van der Helm L., Vandercruyssen G., Mostmans W., Delouvroy F. (2008). 1,5-Benzodiazepines, a novel class of hepatitis C virus polymerase nonnucleoside inhibitors. Antimicrob. Agents Chemother..

[65] Nyanguile O., Devogelaere B., Fanning G.C. (2010). 1a/1bsubtype profiling of nonnucleoside polymerase inhibitors of hepatitis C virus. J. Virol..

[66] Tomei L., Altamura S., Migliaccio G. (2003). Mechanism of action and antiviral activity of benzimidazole-based allosteric inhibitors of the hepatitis C virus RNA-dependent RNA polymerase. J. Virol..

[67] Boyce S.E., Tirunagari N., Niedziela-Majka A., Perry J., Wong M., Kan E., Lagpacan L., Barauskas O., Hung M., Fenaux M. (2014). Structural and regulatory elements of HCV NS5B polymerase—B-Loop and C-terminal tail—Are required for activity of allosteric thumb site II inhibitors. PLoS ONE.

[68] Howe A.Y., Cheng H., Thompson I., Chunduru S.K., Herrmann S. (2006). Molecular mechanism of a thumb domain hepatitis C virus nonnucleoside RNA-dependent RNA polymerase inhibitor. Antimicrob. Agents Chemother..

[69] Davis B., Thorpe I.F. (2013). Molecular simulations illuminate the role of regulatory components of the RNA polymerase from the hepatitis C virus in influencing protein structure and dynamics. Biochemistry.

[70] Ando I., Adachi T., Ogura N., Toyonaga Y., Sugimoto K. (2012). Preclinical characterization of JTK-853, a novel nonnucleoside inhibitor of the hepatitis C virus RNA-dependent RNA polymerase. Antimicrob. Agents Chemother..

[71] Caillet-Saguy C., Simister P.C., Bressanellli S. (2011). An objective asessment of conformational variability in complexes of hepatitis C virus polymerase with non-nucleoside inhibitors. J. Mol. Biol..

[72] Beaulieu P. (2009). Recent advances in the development of NS5B polymerase inhibitors for the treatment of hepatitis C virus infection. Expert Opin. Ther. Pat..

[73] Pflug A., Guilligay D., Reich S., Cusack S. (2014). Structure of influenza a polymerase bound to the viral RNA promoter. Nature.

[74] Reich S., Guilligay D., Pflug A., Malet H., Berger I., Crepin T., Hart D., Lunardi T., Nanao M., Ruigrok R.W. (2014). Structural insight into cap-snatching and RNA synthesis by influenza polymerase. Nature.

